# Evidence-based clinical practice guidelines for gastroesophageal reflux disease 2021

**DOI:** 10.1007/s00535-022-01861-z

**Published:** 2022-02-28

**Authors:** Katsuhiko Iwakiri, Yasuhiro Fujiwara, Noriaki Manabe, Eikichi Ihara, Shiko Kuribayashi, Junichi Akiyama, Takashi Kondo, Hiroshi Yamashita, Norihisa Ishimura, Yuichi Kitasako, Katsunori Iijima, Tomoyuki Koike, Nobuo Omura, Tsutomu Nomura, Osamu Kawamura, Shuichi Ohara, Soji Ozawa, Yoshikazu Kinoshita, Satoshi Mochida, Nobuyuki Enomoto, Tooru Shimosegawa, Kazuhiko Koike

**Affiliations:** 1 Guidelines Committee for Creating and Evaluating the “Evidence-Based Clinical Practice Guidelines for Gastroesophageal Reflux Disease”, The Japanese Society of Gastroenterology, 6F Shimbashi i-MARK Building, 2-6-2 Shimbashi, Minato-ku, Tokyo, 105-0004 Japan; 2 Department of Gastroenterology, Nippon Medical School Graduate School of Medicine, 1-1-5 Sendagi, Bunkyo-ku, Tokyo, 113-8603 Japan

**Keywords:** Guidelines, Gastroesophageal reflux disease (GERD), Reflux esophagitis, Non-erosive reflux disease, Proton pump inhibitor, Potassium-competitive acid blocker (P-CAB), Vonoprazan, Algorithm, Heartburn, Japanese traditional medicine, Prokinetic drug

## Abstract

In Japan, with the increasing prevalence of gastroesophageal reflux disease (GERD) and growing public interest, the Japanese Society of Gastroenterology issued Evidence-based Clinical Practice Guidelines for GERD (1st edition) in 2009 and a revised 2nd edition in 2015. A number of studies on GERD were subsequently conducted in Japan and abroad, and vonoprazan, a potassium-competitive acid blocker (P-CAB), became available for the first time in Japan in February 2015. The revised 3rd edition (Japanese edition), which incorporates new findings and information, was published in April 2021. These guidelines are summarized herein, particularly sections related to the treatment of GERD. The important clinical issues addressed in the present revision are (i) the introduction of treatment algorithms that classify GERD into reflux esophagitis and non-erosive reflux disease, (ii) the clarification of treatment algorithms based on to the severity of reflux esophagitis, and (iii) the positioning of vonoprazan in the treatment for GERD. The present guidelines propose vonoprazan as the initial/maintenance treatment for severe reflux esophagitis. They also recommend vonoprazan or PPI as an initial treatment for mild reflux esophagitis and recommended PPI and proposed vonoprazan as maintenance treatment. These updated guidelines offer the best clinical strategies for GERD patients in Japan and hope that they will be of global use for the diagnosis and treatment for GERD.

## Introduction

Evidence-based clinical practice guidelines for gastroesophageal reflux disease (GERD) 2015 (revised 2nd edition) were published in October 2015 [1]. New findings on the management of GERD were subsequently reported, and vonoprazan, a potassium-competitive acid blocker (P-CAB), became available in Japan in February 2015 for the first time as a treatment for reflux esophagitis (RE) [2]. Since the addition of new information on the management of GERD to the guidelines and decisions on the positioning of vonoprazan for GERD treatment were needed, the guidelines committee of the Japanese Society of Gastroenterology (JSGE) was convened in July 2018 and it was decided to start revising the guidelines for GERD.

The guidelines committee of the JSGE decided to reclassify clinical questions (CQs) in the revised 2nd edition as follows: Background questions (BQs): Questions that were definitively concluded or gained 100% agreement in the previous guidelines. CQs: Questions that affect the course of treatment, and for which recommendations and criteria for the recommendations may be established by exhaustive literature reviews. Future research questions (FRQs): Questions for which recommendations and criteria for the recommendations cannot be established by exhaustive literature reviews (questions that lack sufficient evidence and need to be examined in future).

Clinical practice guidelines are targeted to general clinicians as primary users. The guidelines also aim to provide useful information to medical workers, GERD patients, and their families other than physicians involved in the treatment of GERD. When revising the guidelines, it was agreed as a principle that they were to be consistent with Evidence-based clinical practice guidelines for GERD 2015 (revised 2nd edition).

The basic principles for the preparation of the guidelines were based on the Minds Manual for Clinical Practice Guideline Development 2017 [3] and the JSGE clinical practice guidelines [4]. The quality of evidence was assessed using the Grading of Recommendations Assessment, Development, and Evaluation (GRADE) system [5, 6]. The quality of evidence was graded as A (high), B (moderate), C (low), and D (very low). The recommendation strength was indicated as either a “strong recommendation” or “weak recommendation”. Recommendations were formulated by a modified Delphi technique, with 70% or more votes in agreement.

GERD was classified into erosive and non-erosive GERD in the first and revised 2nd editions; however, the terms RE and non-erosive reflux disease (NERD), which are widely used in daily clinical practice, are used in the 3rd revision.

In the present revision of the guidelines, (i) the introduction of treatment algorithms that classify GERD into RE and NERD, (ii) the clarification of treatment algorithms according to the severity of RE, and (iii) the positioning of vonoprazan in the treatment for GERD were selected as important clinical issues.

CQs and FRQs corresponding to these important clinical issues were created. In addition, CQs in the revised 2nd edition (60 items) were reviewed and those that obtained consensus in the revised 2nd edition were adopted as BQs (52 items). Seventy-one questions consisting of 10 CQs, 9 FRQs, and 52 BQs were established. Among the 71 questions, 2 are related to epidemiology (2 BQs), 8 to pathophysiology, (8 BQs), 11 to diagnosis (10 BQs and 1 FRQ), 16 to medical treatments (6 BQs, 6 CQs, 4 FRQs), 12 to surgical treatments (6 BQs, 2 CQs, and 4 FRQs), 9 to esophagitis after upper gastrointestinal surgery (8 BQs and 1 FRQ), 6 to extra-esophageal symptoms (6 BQs), and 7 to Barrett’s esophagus (6 BQs and 1 CQ). The guidelines were made more exhaustive by the addition of 11 extra questions over the 60 questions in the revised 2nd edition.

After creating CQs, FRQs, and BQs, they were finalized through reviews and modifications by the guideline evaluation committee. Concerning CQs and FRQs, a literature search was performed by the Japan Medical Library Association (review period: 1983–May 2019 for English literature; 1983–June 2019 for Japanese literature), and important studies published outside the search period were added as extra-search period literature. A systematic review was performed for some CQs. Regarding BQs, references were hand-searched by the guidelines committee members.

Concerning CQs, “recommendations” and “comments” were prepared, and the guidelines writing committee assessed the strength and evidence level of the recommendations by deliberation based on the Delphi method. Regarding BQs and FRQs, “statements” and “comments” were prepared.

Since these guidelines target Japanese GERD patients, the guideline creation committee decided to prepare them by placing priority on studies from Japan if there were domestic studies with a high evidence level.

After the final draft of the guidelines was evaluated by the guideline evaluation committee and modified, it was disclosed to the JSGE members, public comments were made, and, through discussions on public comments, the present guidelines were completed.

This manuscript is an English version that mainly focuses on the treatment section of Evidence-based clinical practice guidelines for GERD 2021. It consists of a brief summary of each section of the guidelines, “recommendations” and “comments” on CQs related to diagnosis and treatment, and “statements” and “comments” concerning FRQs. By presenting the algorithms for the diagnosis and treatment of GERD, it aims to disseminate these guidelines worldwide.

## Definitions of terms used in the present guidelines

### GERD

GERD is a condition in which gastroesophageal reflux (GER) causes either esophageal mucosal injuries, annoying symptoms, or both. It is classified into RE with esophageal mucosal injuries and NERD with symptoms alone.

### GER

GER is classified into “acidic GER” and “non-acidic (weakly acidic, alkaline) GER”.

### Proton pump inhibitor (PPI)-resistant GERD

Defined as a condition in which (i) esophageal mucosal injuries do not heal and/or (ii) reflux symptoms considered to be due to GERD are not sufficiently mitigated even after the oral administration of PPI at a standard dose for 8 weeks.

### P-CAB-resistant GERD

Defined as a condition in which (i) esophageal mucosal injuries do not heal and/or (ii) reflux symptoms considered to be caused by GERD are not sufficiently alleviated even after the oral administration of vonoprazan at 20 mg for 4–8 weeks.

### Postoperative esophagitis

Postoperative esophagitis includes esophagitis developed after gastrectomy (including total gastrectomy), esophagectomy, or anti-reflux surgery for GERD, but not after anti-obesity surgery.

### Barrett’s esophagus (BE)

The definition of BE is not currently standardized in Japan or abroad (whether biopsy has been performed, the length of Barrett’s mucosa, and judgments about the esophagogastric junction), and its standardization is required in future. In the present guidelines, the definition “the esophagus with Barrett’s mucosa (a columnar epithelium that extends continuously from the stomach to the esophagus regardless of the presence of intestinal metaplasia)”, by the Japanese Esophageal Society (The Japanese Classification of Esophageal Cancer, 11th ed) was applied.

## Algorithms for the diagnosis and treatment of GERD

**Fig. 1:**
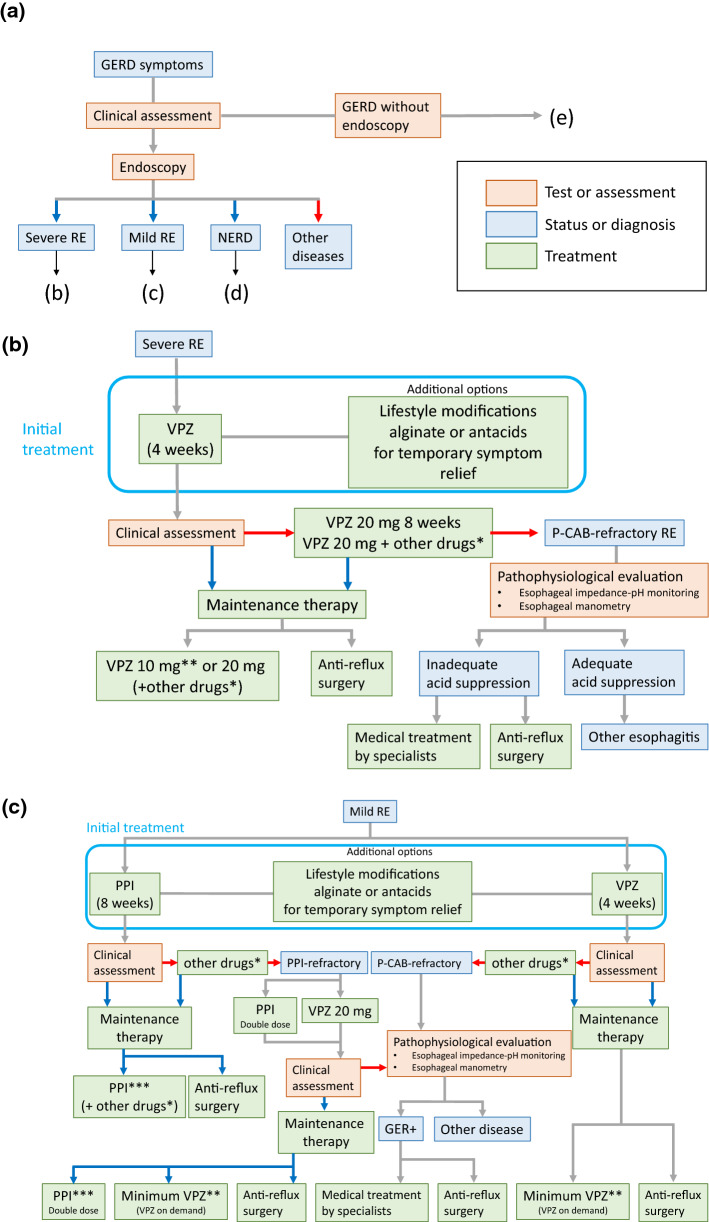

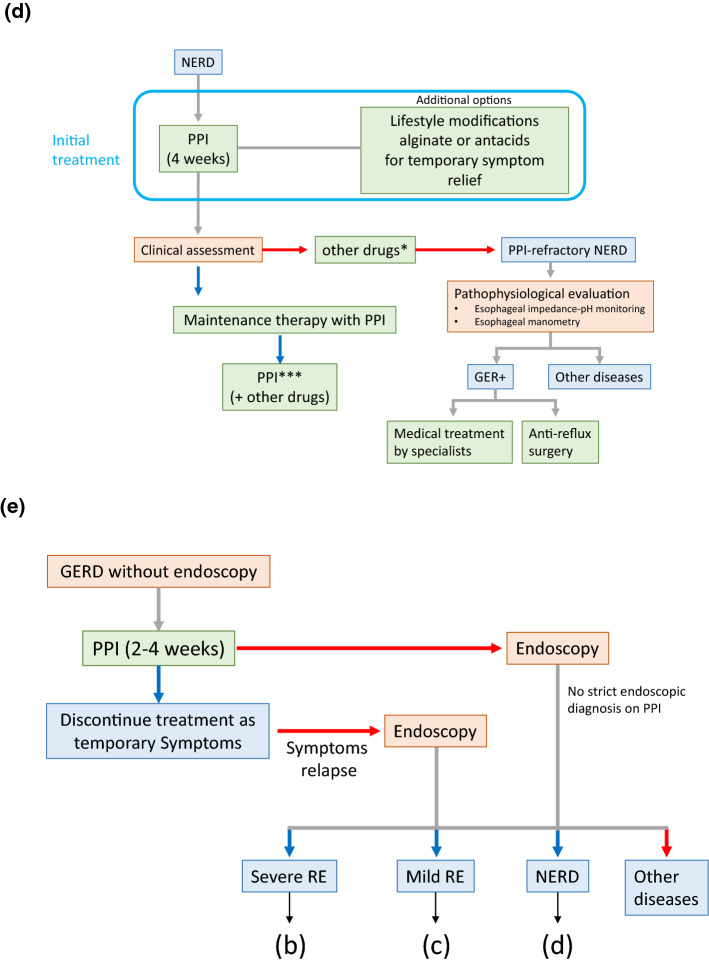
Algorithm for the diagnosis and treatment of gastroesophageal reflux disease (GERD). **a** Diagnosis of GERD with endoscopy. **b** Treatment strategy for severe reflux esophagitis (RE). **c** Treatment strategy for mild RE. **d** Treatment strategy for non-erosive reflux disease (NERD). **e** Diagnosis of GERD without endoscopy. Red arrows: judged to be negative or unsuccessful treatment. Blue arrows: judged to be positive or successful treatment. *Prokinetics or Japanese herbal medicine. **Minimal dose of PPI used in cases with good control during 10 mg of vonoprazan. ***Minimal doses of PPI or on-demand therapy may be used

Figure 1a–e shows algorithms for the diagnosis and treatment of GERD.

**Diagnosis (Fig.** 1**a**): When GERD is suspected based on a clinical assessment, 2 types of algorithms are proposed: (i) endoscopy is initially performed before the administration of PPI, and (ii) the administration of PPI is initiated without endoscopy. In cases on which endoscopy is initially performed, GERD is subdivided into severe RE (grade C or D of Los Angeles (LA) classification), mild RE (grade A or B of LA classification), NERD, and other diseases, and a treatment algorithm (Fig. 1b–d) is proposed for each.

**Treatment for severe RE (Fig. **1**b):** We recommend 20 mg vonoprazan for 4 weeks as an initial treatment. Additional treatments include lifestyle modifications and alginate or antacids for temporary symptom relief (these treatments are also administered to patients with mild RE and NERD). In cases that respond to vonoprazan, affirmative maintenance therapy using 10 or 20 mg vonoprazan, or combination therapy defined as P-CAB with pro-kinetics or Japanese herbal medicine needs to be used to prevent the development of complications. In cases that achieve good control with maintenance therapy of 10 mg vonoprazan, a change to minimal PPI treatment is also possible for maintenance therapy. Anti-reflux surgery also needs to be considered. In cases that do not respond to the initial treatment, vonoprazan 20 mg may be continued for up to 8 weeks or combination therapy may be initiated.

**Treatment for mild RE (Fig. **1**c):** We recommend a standard dose of PPI or 20 mg vonoprazan therapy as an initial treatment. A minimal dose of PPI or P-CAB therapy (including on-demand therapy), or combination therapy may be selected as maintenance therapy. Anti-reflux surgery also needs to be considered. As a therapeutic strategy for PPI-resistant mild RE, we recommend a double dose of PPI or 20 mg vonoprazan.

**Treatment for NERD (Fig. **1**d):** We recommend PPI for 4 weeks as an initial treatment, and a minimal dose of PPI (including on-demand therapy) or combination therapy to improve symptoms may be selected as maintenance therapy.

**Treatment for GERD without endoscopy (Fig. **1**e):** The PPI test may be applied for cases without endoscopy. In cases in which symptomatic resolution is achieved with PPI before endoscopy, they are transient symptoms and treatment may be discontinued. If symptoms persist or relapse, endoscopy needs to be performed. No strict endoscopic diagnosis is possible under or after PPI treatment.

**Treatment for refractory cases (Fig. **1**b–d):** In cases of P-CAB- resistant RE (Fig. 1b, c), double-dose PPI-resistant mild RE (Fig. 1c), or PPI-resistant NERD (Fig. 1d), a pathophysiological evaluation by multichannel intraluminal impedance-pH monitoring and/or esophageal manometry is recommended to examine the relationship between symptoms and the esophageal pathophysiology or the status of acid suppression. If a relationship between GER and symptoms is established or inadequate acid suppression on P-CAB in severe RE is present, medical treatment by an expert or surgery is an alternative.

## Summary of epidemiology

The prevalence of GERD has been increasing since the end of the 1990s due to the enhanced secretion of gastric acid, a decrease in the *Helicobacter pylori* infection rate, and a westernized lifestyle [7]. The prevalence of RE is estimated to be 10% in the general adult population [7]. An advanced age and severe RE have been associated with esophageal stenosis and bleeding [8].

## Summary of pathophysiology

The excessive exposure of the esophagus to gastric acid due to GER is a major cause of esophageal mucosal injury, the extent of which increases with the severity of RE [9–12]. The following mechanisms contribute to the development of acidic GER: transient lower esophageal sphincter (LES) relaxation, increased abdominal pressure, and low LES pressure [9, 13–21]. In addition, esophageal hiatal hernia results in increased acid reflux and delayed acid clearance in the esophagus, leading to excessive esophageal gastric acid exposure. Furthermore, esophageal motility disorders expose the esophagus to excessive gastric acid [20, 22–27]. With the development of multichannel intraluminal impedance-pH monitoring, it has become possible to detect acidic and non-acidic GER with high sensitivity. Previous studies using this method have shown that non-acidic GER is an etiology of GERD [28, 29]. The pathogenesis of NERD is not necessarily the same as that of RE [30–35]. In the Rome IV criteria revised in 2016, diseases presenting with heartburn, such as GERD, were classified into four categories: erosive esophagitis, NERD, reflux hypersensitivity, and functional heartburn, based on esophageal hypersensitivity and acid exposure [36]. Of these, NERD in clinical practice includes (i) true NERD caused by abnormal esophageal acid exposure, similar to RE, (ii) reflux hypersensitivity without abnormal esophageal acid exposure, but with increased esophageal sensitivity and symptoms caused by small amounts of acid or non-acidic GER, and (iii) functional heartburn with symptoms unrelated to GER.

## Summary of diagnosis

GERD is generally diagnosed by a combination of clinical symptoms, objective testing with endoscopy, reflux monitoring, and responses to anti-secretory therapy.

GERD typically manifests as heartburn and regurgitation, but may also present with atypical symptoms (non-cardiac chest pain or extra-esophageal symptoms) [37]. A wide variety of self-administered questionnaires has been developed for the assessment of reflux symptoms to establish the diagnosis of GERD and measure responses to treatment [38]. The severity of GERD symptoms does not always correlate with the endoscopic severity of mucosal injury [39].

The Los Angeles classification is the most validated, reproducible, and accurate system to describe the endoscopic appearance of RE and grade its severity [11]. Several novel image-enhanced endoscopy techniques have been shown to improve the detection of minimal changes.

Ambulatory esophageal reflux monitoring (pH or impedance-pH) is the only test that allows the presence of an esophageal reflux burden and/or a relationship between symptoms and reflux episodes to be confirmed [40].

The PPI test is a pragmatic approach in clinical practice due to its limited invasiveness, lower cost, and symptomatic response corroborating a clinical suspicion of GERD [41]. It is a useful diagnostic test for patients presenting with typical reflux symptoms or chest pain [42], but is of limited value for those with extra-esophageal manifestations [43–45].

Refractoriness to PPI may be related to reflux or non-reflux causes. The latter includes functional heartburn, eosinophilic esophagitis, esophageal motility disorders, and phycological comorbidity [46].

**FRQ-1: Is the P-CAB test more useful than the PPI test?**

- The P-CAB test may be more useful than the PPI test.

*Comment*: Empirical acid suppression with the “PPI test” or “P-CAB test” is often used in the primary care setting as a simple, non-invasive, and cost-saving ‘diagnostic’ test to evaluate whether upper gastrointestinal symptoms are due to GERD. However, a meta-analysis evaluating the diagnostic test characteristics of PPI treatment suggested some limitations to this approach. The pooled sensitivity and specificity of a positive PPI test result were reported to be 0.78 and 0.54, respectively, when an abnormal 24-h pH study was used as the reference standard. The mechanisms of a false-positive test include a non-GERD etiology (such as dyspepsia), the placebo effect, and esophageal hypersensitivity to acid, while those of a false-negative test include a suboptimal dosage and the duration of PPI treatment to ameliorate symptoms [41].

P-CAB achieves the rapid and marked suppression of gastric acid secretion in a dose-dependent manner [47, 48], which results in greater symptom improvements than conventional PPI in patients with RE and NERD [49–51]. Therefore, the P-CAB test may be more useful than the PPI test; however, further investigations are warranted to assess the optimal dosage and duration of P-CAB as well as the appropriate tool for evaluating symptomatic relief.

## Summary of medical treatment

This revision of the guidelines clarifies three important clinical questions in the treatment of GERD; the first is the introduction of a separate algorithm for RE and NERD, the second is clarification of the treatment algorithm according to the endoscopic severity of RE, and the third is clarification of the position of the new gastric acid suppressant, P-CAB and conventional PPI. The initial treatment of GERD consists of PPI for NERD [1], PPI or P-CAB for mild RE [52], and P-CAB for severe RE [52], along with lifestyle modifications and sodium alginate/antacids [53]. Regarding cases refractory to PPI, not only a change to P-CAB, but also combination therapy with Japanese herbal medicines [54] and/or prokinetic drugs are recommended [55]. In addition, various functional tests are recommended for more intractable cases to elucidate the underlying pathophysiology [56].

**CQ-1: What needs to be recommended for the initial treatment of mild RE** **,** **PPI, or P-CAB?**

- PPI and P-CAB both achieve esophageal mucosal healing in the initial treatment of mild RE. Both medications are recommended as a first-line treatment for patients with mild RE. (Strong recommendation, Evidence level B, 100% agreed).

**Fig. 2:**
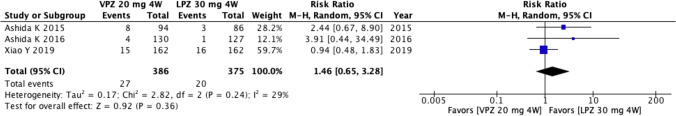
Comparison of the non-healing rate for mild reflux esophagitis between vonoprazan (VPZ) at 20 mg once daily for 4 weeks and lansoprazole (LPZ) at 30 mg once daily for 4 weeks by a meta-analysis

**Fig. 3:**
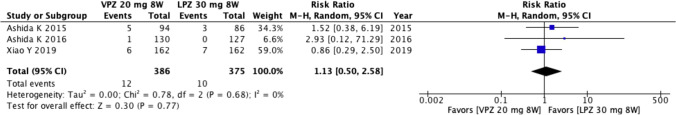
Comparison of the non-healing rate for mild reflux esophagitis between vonoprazan (VPZ) at 20 mg once daily for 8 weeks and lansoprazole (LPZ) at 30 mg once daily for 8 weeks by a meta-analysis

**Fig. 4:**
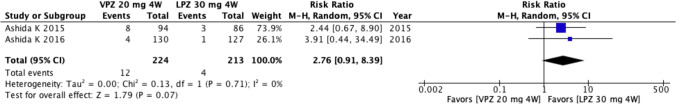
Comparison of the non-healing rate of mucosal injury (damage) between vonoprazan (VPZ) at 20 mg once daily for 4 weeks and lansoprazole (LPZ) at 30 and 20 mg once daily for 4 weeks by a meta-analysis limited to two domestic studies

**Fig. 5:**
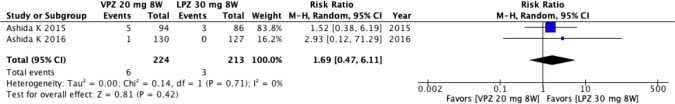
Comparison of the non-healing rate of mucosal damage between vonoprazan (VPZ) at 20 mg once daily for 8 weeks and lansoprazole (LPZ) at 30 mg once daily for 8 weeks by a meta-analysis limited to two domestic studies

**Fig. 6:**
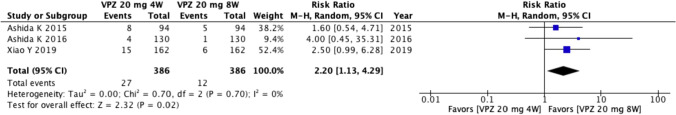
Comparison of the non-healing rate of mucosal injury between vonoprazan (VPZ) at 20 mg once daily for 4 and 8 weeks by a meta-analysis

**Fig. 7:**
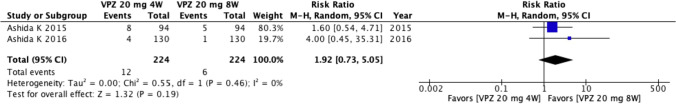
Comparison of the non-healing rate of mucosal injury between vonoprazan (VPZ) at 20 mg once daily for 4 and 8 weeks by a meta-analysis limited to two domestic studies

**Fig. 8:**
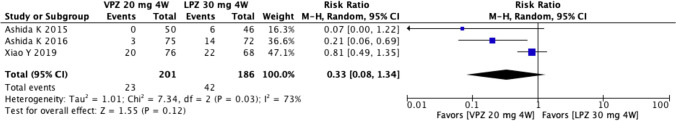
Comparison of the non-healing rate of severe reflux esophagitis between vonoprazan (VPZ) at 20 mg once daily for 4 weeks and lansoprazole (LPZ) at 30 mg once daily for 4 weeks by a meta-analysis

*Comment*: Initial treatment is defined as treatment within 8 weeks. A systematic review of clinical trials comparing conventional PPI with P-CAB that are currently available in Japan was performed. Two domestic double-blinded RCTs compared the treatment efficacy of lansoprazole at 30 mg once daily and vonoprazan at 20 mg once daily [2, 52]. Out of the search range, there was one additional overseas study in the above-described setting [57]. A meta-analysis was conducted to compare the non-healing rate of mucosal injury after the initial treatment between lansoprazole at 30 mg once daily and vonoprazan at 20 mg once daily. In comparisons with lansoprazole, vonoprazan did not reduce the non-healing rate of mucosal injury for 4 weeks (risk ratio (RR) = 1.46, 95% CI [0.65–3.28]) (Figs. 2) or 8 weeks (RR = 1.13, 95% CI [0.50–2.58]) (Figs. 3), and a meta-analysis, excluding one overseas RCT, showed that vonoprazan did not affect the rate for 4 weeks (RR 2.76, 95% CI [0.91–8.39]) (Fig. 4) or 8 weeks (RR 1.69, 95% CI [0.47–6.11]) (Fig. 5). In contrast, vonoprazan at 20 mg once daily for 4 weeks significantly increased the non-healing rate of mucosal injury over that with vonoprazan at 20 mg once daily for 8 weeks (RR 2.20, 95% CI [1.13–4.29]) (Fig. 6), whereas it did not increase the rate in a meta-analysis limited to two domestic RCTs (RR 1.92, 95% CI [0.73–5.05]) (Fig. 7). Although neither treatment-emergent adverse events nor symptom improvements were exclusively set as the outcome for the meta-analysis in cases of mild RE, no significant differences were observed in treatment-emergent adverse events between lansoprazole and vonoprazan in patients with mild and severe RE [2, 52, 57]. In contrast, a double-blind RCT demonstrated that vonoprazan at 20 mg once daily achieved more rapid improvements in heartburn in patients with mild and severe RE than lansoprazole at 30 mg once daily [49]. A double-blind RCT has not yet been conducted to compare the effects of vonoprazan in patients with RE to those of PPI other than lansoprazole.

**CQ-2: What needs to be recommended for the initial treatment of severe RE, PPI, or P-CAB?**

- Vonoprazan at 20 mg once daily for 4 weeks is proposed as the initial treatment of patients with severe RE. (Weak recommendation, Evidence level: C, 100% agreed)

**Fig. 9:**
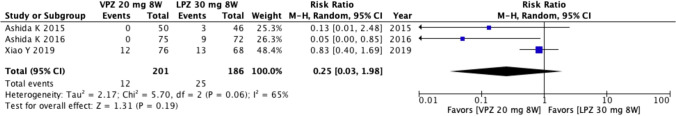
Comparison of the non-healing rate of severe reflux esophagitis between vonoprazan (VPZ) at 20 mg once daily for 8 weeks and lansoprazole (LPZ) at 30 mg once daily for 8 weeks by a meta-analysis

**Fig. 10:**
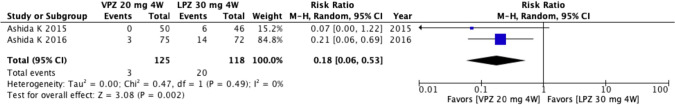
Comparison of the non-healing rate of severe reflux esophagitis between vonoprazan (VPZ) at 20 mg once daily for 4 weeks and lansoprazole (LPZ) at 30 mg once daily for 4 weeks by a meta-analysis limited to two domestic studies

**Fig. 11:**
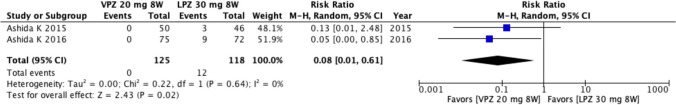
Comparison of the non-healing rate of severe reflux esophagitis between vonoprazan (VPZ) at 20 mg once daily for 8 weeks and lansoprazole (LPZ) at 30 mg once daily for 8 weeks by a meta-analysis limited to two domestic studies

**Fig. 12:**
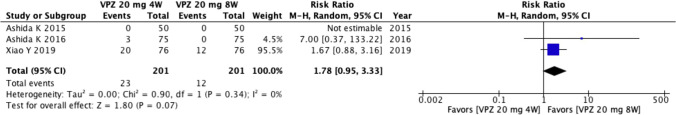
Comparison of the non-healing rate of severe reflux esophagitis between vonoprazan (VPZ) at 20 mg once daily for 4 and 8 weeks by a meta-analysis

**Fig. 13:**
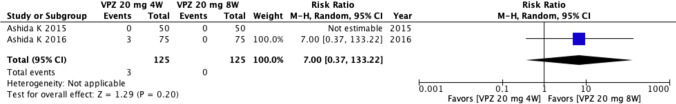
Comparison of the non-healing rate of severe reflux esophagitis between vonoprazan (VPZ) at 20 mg once daily for 4 and 8 weeks by a meta-analysis limited to two domestic studies

*Comment*: A systematic review of clinical trials comparing conventional PPI with P-CAB that are currently available in Japan was performed. The primary outcome was the non-healing rate of RE, and the secondary outcomes were the rate of treatment-emergent adverse events and cost-effectiveness. Three double-blinded RCTs were included in meta-analyses: two RCTs from Japan [2, 52] and one RCT from Asia [57]. The treatment efficacies of lansoprazole at 30 mg once daily and vonoprazan at 20 mg once daily were compared in these RCTs. In comparisons with lansoprazole, vonoprazan did not reduce the non-healing rate of RE after treatment for 4 weeks (RR 0.33, 95% CI [0.08–1.34]) (Fig. 8) or 8 weeks (RR 0.25, [0.03–1.98]) (Fig. 9). However, when the foreign RCT was excluded, the non-healing rate of RE was significantly lower with vonoprazan than with lansoprazole after their administration for 4 weeks (RR 0.18, 95% CI [0.06–0.53]) (Fig. 10) and 8 weeks (RR 0.08, 95% CI [0.01–0.61]) (Fig. 11). In addition, in comparisons with lansoprazole for 8 weeks, vonoprazan administered for 4 weeks had a significantly lower non-healing rate of RE (RR 0.28, 95% CI [0.09–0.89]). The non-healing rate of RE after the administration of vonoprazan for 4 weeks was not significantly different from that of vonoprazan administered for 8 weeks in studies conducted both inside and outside of Japan (RR 1.78, 95% CI [0.95–3.33]) (Fig. 12), as well as in studies conducted only in Japan (RR 7.00, 95% CI [0.37–133.22]) (Fig. 13). The rates of treatment-emergent adverse events were not significantly different between vonoprazan and lansoprazole.

A meta-analysis using a network analysis showed that vonoprazan at 20 mg once daily had a significantly higher healing rate for severe RE than lansoprazole at 30 mg once daily, omeprazole at 20 mg once daily, esomeprazole at 20 mg once daily, and rabeprazole at 20 mg once daily [58]. Although vonoprazan at 20 mg once daily had a similar healing rate for severe RE to rabeprazole at 10 mg twice a day and rabeprazole at 20 mg twice a day, these doses of rabeprazole cannot be used as an initial treatment in the Japanese health care system. Regarding cost-effectiveness, vonoprazan at 20 mg once daily for 4 weeks as an initial treatment for severe RE was significantly more cost-effective than esomeprazole at 20 mg once daily or rabeprazole at 10 mg once daily for 8 weeks [59].

**CQ-3: What approach is needed when the effect of a PPI is insufficient at the standard dose?**

- If esophageal mucosal breaks do not heal or the patient develops severe symptoms despite standard PPI treatment, a change to the standard dose of PPI twice daily or vonoprazan 20 mg once daily is recommended. (Strong recommendation, Evidence level B, 93% agreed)
- Regarding those who do not respond to standard PPI treatment, there are options to switch to another PPI or add the prokinetic drug, mosapride or the traditional Japanese herbal medicine, rikkunshito. (Weak recommendation, Evidence level C, 86% agreed)

*Comment*: PPI has exhibited high efficacy in the treatment of GERD and is now widely used in clinical practice. However, some patients with GERD are resistant to PPI and have a lower quality of life and impaired labor productivity [60]. Regarding the treatment of PPI-resistant GERD, several multicenter studies have shown that increasing the dose of PPI [61], changing the type of PPI [62], switching to vonoprazan [63], adding rikkunshito [54], or adding mosapride or baclofen [64] improved symptoms in some patients.

Since more patients with NERD are considered to be resistant to PPI than those with RE, its clinical management is particularly important [65]. Since the degree of acidic GER in the esophagus in NERD is between that of healthy individuals and mild RE [66], its treatment requires the addition of other medications to PPI. Among patients with NERD with no improvement in symptoms with PPI therapy, improvements have so far been reported in some patients with increased doses of PPI [67] or the additional administration of mosapride [68], acotiamide [69], or rikkunshito [70]. Due to the wide range of conditions that mimic NERD, functional tests, including multichannel intraluminal impedance-pH monitoring, are recommended for patients with NERD who are refractory to adequate treatment, as described above.

**CQ-4: Which is recommended for the long-term management of mild RE, PPI, or P-CAB?**

- PPI is recommended for the long-term maintenance of mild RE. (Strong recommendation, Evidence level C, 100% agreed)
- P-CAB is proposed for the long-term maintenance of mild RE. (Weak recommendation, Evidence level C, 86% agreed)

*Comment*: A double-blind Japanese phase III study on 24-week maintenance treatment for RE with vonoprazan showed that the 10-mg and 20-mg vonoprazan groups both had significantly lower rates of recurrent RE in endoscopic examinations than the 15-mg lansoprazole group (vonoprazan 10 mg, vonoprazan 20 mg, and lansoprazole 15 mg; 5.1, 2.0, and 16.8%, respectively) [71]. Furthermore, in comparisons of recurrence rates in patients with Los Angeles classification Grade A or B, recurrence was detected in 11.0% of patients in the lansoprazole 15-mg group, while 3.1% of patients in the vonoprazan 10 mg group and 1.3% of patients in the vonoprazan 20 mg group showed a further reduction in the recurrence rate. Furthermore, a network meta-analysis of maintenance therapy for RE with PPI and vonoprazan showed that the effects of maintenance therapy with 10 mg vonoprazan were similar to or better than those of PPI [72].

On the other hand, on-demand therapy, which is therapy that patients take “as needed”, is reportedly useful as long-term maintenance therapy for mild RE [73]. A non-randomized, open-label, comparative study on on-demand therapy with 20 mg vonoprazan in patients with mild RE who had been well maintained on PPI was conducted in Japan [74]. The endoscopic remission rate after 6 months of on-demand therapy was 86.2% and no significant differences were observed in the degree of overall satisfaction between PPI and on-demand maintenance therapy. Furthermore, the time to the resolution of reflux symptoms was shorter with 20 mg vonoprazan than with 30 mg lansoprazole [49]. Therefore, vonoprazan may be more suitable than conventional PPI in on-demand therapy, which requires a rapid onset of action. However, since there is currently insufficient information on the safety of the long-term administration of vonoprazan, careful observations are required during the long-term administration of vonoprazan. Based on the above findings, the guidelines recommend PPI and propose vonoprazan as long-term maintenance therapy for mild RE.

**CQ-5: Which is recommended for the long-term management of severe RE,** **PPI, or P-CAB?**

- Vonoprazan at 10 mg once daily is proposed for the long-term management of severe RE due to the low endoscopic relapse rate. (Weak recommendation, Evidence level C, 93% agreed)

*Comment*: Since severe RE has a higher acid reflux rate than mild RE [12], the recurrence of esophageal mucosal injury is expected without maintenance therapy [75]. Severe RE is also associated with a high risk of bleeding and stenosis (grade C odds ratio 15.38; 95% CI 8.62–28.37, grade D odds ratio: 71.49; 95% CI 37.47–142.01) [8]. Therefore, continuous gastric acid suppression is necessary for long-term management.

However, the endoscopic relapse rates of maintenance treatment for severe RE were 27% after 104 weeks of 10 mg rabeprazole [76], 24% after 24 weeks of 20 mg esomeprazole [77], and 26% after 52 weeks of rabeprazole at 10 mg twice daily in patients with standard-dose PPI-resistant RE [78]. Furthermore, during PPI maintenance therapy for severe RE, complications, such as bleeding and stenosis, were observed in approximately 20% of patients [79]. Based on these findings, a low endoscopic relapse rate is desired in the long-term management of severe RE from the viewpoint of preventing complications.

On the 7th day of administration of 10 mg vonoprazan, the gastric pH > 4 holding time rate was 63% [48], which was higher than the standard dose of conventional PPI. The endoscopic relapse rate has not yet been compared between standard-dose PPI and 10 mg vonoprazan for severe RE. A RCT that evaluated endoscopic relapse rates after 24 weeks of 15 mg lansoprazole (half dose), 10 mg vonoprazan, and 20 mg vonoprazan in a sub-analysis of severe RE reported relapse rates of 39.0, 13.2% (*p* = 0.0114), and 4.7% (*p* = 0.0001), respectively [71]. No significant differences were observed in endoscopic relapse rates between 10 and 20 mg vonoprazan, and no severe adverse events occurred. Based on these findings, 10 mg vonoprazan, which has a lower endoscopic relapse rate than 15 mg lansoprazole, is proposed for the long-term management of severe RE; however, direct comparisons between standard-dose PPI and 10 mg vonoprazan are needed. Furthermore, careful follow-ups are required because the adverse events of the long-term administration of 10 mg vonoprazan currently remain unknown.

**CQ-6: Is long-term PPI therapy safe for the treatment of GERD?**

- Long-term PPI therapy is generally safe; however, careful observations are required. (Weak recommendation, Evidence level B, 93% agreed)

*Comment*: Although PPI has shown excellent efficacy in the treatment of acid-related diseases, such as GERD, and has been highly recommended, several concerns have been expressed regarding their long-term administration [80]. However, the Clinical Practice Guidelines 2017 proposed by the American Gastroenterological Association recently reported that the quality of evidence for PPI-related adverse events in these observational studies, crossover studies, or RCTs was low due to the possible effects of residual confounders, consistency issues, and differences in findings between observational studies and RCTs [81]. In addition, a comparison of the clinical efficacy of laparoscopic anti-reflux surgery and omeprazole in the treatment of GERD (the SOPRAN study) and a comparison of the clinical efficacy of laparoscopic anti-reflux surgery and esomeprazole (the LOTUS study) demonstrated the safety associated with long-term maintenance therapy with PPI [82]. Based on these findings, a therapeutic strategy for GERD with PPI needs to be established by balancing their benefits and risks; however, there are generally more benefits. Therefore, when treating GERD with PPI, their dosage and administration needs to be as low and short, respectively, as possible; however, long-term maintenance therapy is recommended with careful attention for cases requiring this treatment. The following is a list of current concerns associated with long-term maintenance therapy with PPI.

## Development of carcinoid tumors

The long-term administration of PPI is currently not expected to exert positive effects on carcinoid tumors, but some caution will be warranted in future.

## Influence on gastrointestinal infections and intestinal bacteria

The use of PPI may slightly increase the risk of intestinal infections. Patients taking PPI are also at risk of dysbiosis, which causes gas-related symptoms and NSAID-induced intestinal damage, as well as increased small intestinal bacterial abnormalities [83]. Therefore, caution is needed in future.

## Drug interactions

Interactions between PPI and other drugs require constant attention, particularly in the elderly who are more likely to take multiple medications. Interactions with diazepam, warfarin, phenytoin, and methotrexate need to be considered [84].

**FRQ-2: What is recommended as the initial treatment for NERD** **,** **PPI, or P-CAB?**

- NERD is classified into (i) NERD with excessive esophageal acid exposure, (ii) esophageal reflux hypersensitivity that presents with reflux symptoms due to increased esophageal sensitivity despite a normal esophageal acid exposure time, and (iii) functional heartburn presenting with symptoms unrelated to reflux, and P-CAB and PPI may both be effective for NERD with excessive esophageal acid exposure.

*Comment*: Evidence-based clinical practice guidelines for GERD (2nd edition) recommend PPI as the first-line treatment for NERD [1]; however, their effectiveness is approximately 50%. Previous studies that evaluated the etiology of PPI-resistant NERD [85–88] suggested that the primary cause of the symptoms of PPI-resistant NERD was unrelated to acid reflux. In 2015, vonoprazan, which suppresses acid secretion more potently than PPI, was approved in Japan. Although there is currently no evidence to support the effectiveness of vonoprazan for NERD [50, 51], it may be effective for NERD with excessive esophageal acid exposure. In future, the answer to this FRQ will be clarified by evaluations of the usefulness of PPI and vonoprazan for the management of NERD with excessive esophageal acid exposure.

**FRQ-3: Is PPI or P-CAB recommended for intermittent therapy or on-demand therapy in the long-term management of NERD that responded to the initial treatment?**

- P-CAB and PPI may be useful for the long-term management of NERD that responds to the initial treatment.

*Comment*: In Evidence-based clinical practice guidelines for GERD 2015 (2nd edition), the first-line treatment for NERD is PPI; however, PPI are only effective in approximately 50% of patients [1]. The main causes of symptoms in the remaining 50% of patients are considered to include conditions other than acidic GER, esophageal motility disorders, eosinophilic esophagitis, and functional heartburn [89]. If PPI are effective, the cause of symptoms of NERD patients is acidic GER. Since NERD is a reflux disease without esophageal mucosal injuries, the control of reflux symptoms is crucial for its treatment.

Continued PPI therapy, which is intermittent PPI therapy in symptomatic periods, is the mainstream maintenance therapy for NERD patients. Regarding on-demand therapy with PPI for NERD, a systematic review and a meta-analysis showed the effectiveness of on-demand therapy with PPI [90] and the effects of reducing medical cost due to a decrease in the number of PPI administration [91].

The use of vonoprazan for NERD is not covered by the national health insurance system in Japan because there is no evidence to support its effectiveness for NERD [50, 51]; however, vonoprazan is considered to be effective for NERD patients with symptoms caused by acidic GER. A previous study employed vonoprazan on-demand therapy for NERD patients [92]. The findings obtained showed that NERD patients who were satisfied with PPI maintenance therapy took vonoprazan at 20 mg as on-demand therapy when they experienced reflux symptoms based on their own judgment. The degree of satisfaction with vonoprazan on-demand therapy was similar to that with PPI maintenance therapy, and the median number of 20-mg vonoprazan tablets taken during the 8-week period was 11 (total number of tablets taken: 3–28). Approximately 30% of patients continued to take 2 or more tablets/week, and this approach was also reported to be advantageous from the viewpoint of medical economics. The answer to this FRQ will be clarified in future by evaluating the effectiveness of intermittent therapy and on-demand therapy using PPI and vonoprazan in NERD patients who responded to acid suppression therapy as the initial treatment.

## Summary of surgical treatment

Indications for surgical treatment are PPI-resistant GERD, the need for long-term maintenance therapy with PPI, and extra-esophageal manifestations, such as asthma, hoarseness, cough, chest pain, and aspiration caused by GER [93, 94]. The long-term outcomes of anti-reflux surgery were found to be satisfactory for typical GERD symptoms, such as heartburn and regurgitation; however, it is not superior to PPI treatment.

**CQ-7: Is surgical treatment useful for drug treatment-resistant RE?**

- Anti-reflux surgery is proposed as an effective treatment for patients with PPI-resistant RE. (Weak recommendation, Evidence level B, 100% agreed)

*Comment*: Few RCTs have compared clinical outcomes between surgical and medical treatments only for patients with PPI-resistant RE. In 2019, a multicenter RCT on genuine PPI-refractory heartburn was conducted by Spechler and colleagues [95]. This study examined patients who continued to have persistent heartburn even after taking 20 mg of omeprazole twice daily for 2 weeks. In this RCT, patients with reflux-related heartburn were strictly defined based on multichannel intraluminal impedance-pH monitoring. They found that the incidence of treatment success with laparoscopic Nissen fundoplication was significantly higher than that with active medical treatment (omeprazole plus baclofen, with the addition of desipramine depending on symptoms) or control medical treatment (omeprazole plus placebo).

The answer to this CQ is limited by the paucity of data on surgical outcomes only in patients with erosive RE who do not heal after taking a regular dose of PPI for 6 to 8 weeks. In addition, the superiority or inferiority of PPI + α treatment (including prokinetic drugs, mucosal protective drugs, and Japanese herbal medicine) to surgical treatment warrants further study by RCTs.

**CQ-8: Is surgical treatment useful for drug treatment-resistant NERD?**

- Anti-reflux surgery is proposed as an effective treatment for PPI-resistant NERD patients if a causal relationship between symptoms and GER is proven by multichannel intraluminal impedance-pH monitoring. (Weak recommendation, Evidence level C, 93% agreed)

*Comment*: Multichannel intraluminal impedance-pH monitoring has recently become available. It provides more detailed information on the pathophysiology of GERD, which allows for patients with pathological reflux to be distinguished from those with functional heartburn; therefore, it is possible to objectively select surgical indications for NERD patients. Anti-reflux surgery is a surgical procedure that reconstructs the GER prevention mechanism, which presumably controls any back flow from the stomach to the esophagus regardless of the properties of the gastric contents. Therefore, surgical treatment is expected to be therapeutically effective for NERD patients if the relationship between symptoms and GER is significant. The treatment outcomes of anti-reflux surgery for NERD patients are reportedly good [96–99] with a symptomatic improvement rate of 80% or more, which is a similar success rate to that for GERD patients who undergo anti-reflux procedures. However, it may be difficult to discuss the real outcomes of surgical treatment for PPI-resistant NERD because the history of PPI administration was not described in detail in previous studies and patient selection criteria for drug-refractory NERD are obscure. Therefore, candidates for anti-reflux surgery need to be very carefully selected among patients with PPI-resistant NERD. Surgical treatment is regarded as a possible treatment option if GER is confirmed by multichannel intraluminal impedance-pH monitoring and it is clear that GER is triggering the onset of symptoms.

## Summary of postoperative esophagitis

Postoperative esophagitis mainly includes esophagitis that developed after gastrectomy (including total gastrectomy) and esophagectomy. Its development is affected by the size of the remnant stomach, the position of the anastomosis, and the reconstruction method [100, 101]. Postoperative esophagitis after total gastrectomy is caused by the duodenal contents (pancreatic juice and bile); however, in surgical procedures with the residual stomach, gastric juice and duodenal juice may both be the cause. As drug therapy, not only acid-suppressive drugs, but also prokinetic agents, protease inhibitors, and mucosal protective agents may be useful. In addition, surgical treatment, particularly the Roux-en-Y procedure, may be useful for the treatment of postoperative esophagitis [102], and we suggest the importance of considering surgical treatment. There is currently no information on the usefulness of lifestyle guidance in the treatment of postoperative esophagitis. In recent years, the number of patients who have undergone proximal gastrectomy has increased. Therefore, it is useful to add an anti-reflux procedure to prevent postoperative esophagitis.

**CQ-9: Is fundoplication useful for the prevention of postoperative esophagitis in reconstruction by the esophago-remnant gastric anastomosis after proximal gastrectomy?**

- The addition of an anti-reflux procedure to prevent postoperative esophagitis is proposed for the esophago-remnant gastric anastomosis after proximal gastrectomy. (Weak recommendation, Evidence level C, 100% agreed)

*Comment*: Postoperative RE is more likely to occur after proximal gastrectomy than after total gastrectomy, and a reconstructive procedure aimed at preventing reflux has been devised. The incidence of esophagitis by the jejunal interposition method is as low as 1.7%, and good long-term results have been reported [103]. On the other hand, since esophagitis frequently occurs in the esophago-remnant gastric anastomosis, the addition of an anti-reflux procedure is required in these cases.

The incidence of postoperative esophagitis does not significantly differ between Toupet-like fundoplication, which involves wrapping the remnant stomach around the esophagus, the double tract method [104], and jejunal interposition [105]. The double flap method (Kamikawa method) has also been reported to be useful, and has recently been performed laparoscopically. The incidence of esophagitis 12 months after surgery is 2.3–5.3%, showing satisfactory outcomes [106]. Furthermore, in comparisons of fundoplication with the double flap method in laparoscopic proximal gastrectomy, the double flap method was significantly more effective at preventing regurgitation and required less PPI after surgery [107].

Although the frequency of complications is within the permissible range, the double flap method is difficult to perform, particularly laparoscopically, and there are difficulties associated with its introduction at this point to more facilities.

Previous studies reported the effects of His angle formation [108] other than fundoplication and the double flap method; however, the number of cases was small and RCTs have not yet been conducted. Accordingly, there is insufficient evidence to recommend this CQ.

## Summary of atypical or extra-esophageal symptoms

GER causes not only typical symptoms (heartburn and regurgitation), but also atypical symptoms, including chest pain, and extra-esophageal manifestations, such as chronic cough, bronchial asthma, laryngitis, sleep disturbance, and dental erosion [37, 109]. The reported prevalence of the extra-esophageal manifestations of GERD markedly varies depending on the group of patients examined as well as its definition [110, 111]. Furthermore, the pathophysiology of these manifestations has not yet been elucidated in detail and frequently have a multifactorial etiology, which suggests that GER may be a co-factor rather than a direct cause of GERD.

Current diagnostic modalities, including esophagogastroduodenoscopy, laryngoscopy, and impedance-pH monitoring, are limited by poor sensitivity and specificity for the diagnosis of these extra-esophageal manifestations. Although acid suppression therapy appears to be effective in patients with GER-related extra-esophageal symptoms, PPIs have not exhibited clear therapeutic benefits in the treatment of these symptoms [112]. Further studies are needed to establish the diagnosis of and treatment strategies for the extra-esophageal manifestations of GERD.

## Summary of BE

In Japan, BE is defined as the columnar-lined esophagus that extends continuously from the stomach to the esophagus, with or without intestinal metaplasia [113]. Globally, the definition of BE, namely, the necessity of biopsy (histological criteria), length, and differences in the endoscopic diagnosis of the esophagogastric junction, is not unified [114]. In addition to the intra-esophageal reflux of gastric acid and bile acid [115, 116] high concentrations of nitric oxide generated locally at the human esophagogastric junction [117, 118] may be a cause of BE.

BE is described as “long-segment BE (LSBE)” when the circumferential length of BE is 3 cm or more, and “short segment BE (SSBE)” when BE is less than 3 cm or non-circumferential [113]. The average frequency of LSBE among the general Japanese population is 0.3%, which is markedly lower than that of SSBE (15.8%).

The carcinogenic risk of BE is strongly associated with its length [119, 120]. In a multicenter prospective observational study conducted in Japan [121], the cancer incidence of LSBE was 1.2% per year, which was similar to that in Western countries [119, 120]. Therefore, LSBE may be a target for surveillance, but accounts for only a small portion of BE in Japan. Since the cancer incidence of SSBE is currently unknown, the necessity for surveillance remains unknown in Japan.

**CQ-10: Is medication useful for preventing the carcinogenesis of BE?**

- Although the administration of high-dose PPI may effectively prevent the carcinogenesis of BE, medication that prevents the carcinogenesis of BE is not currently used in Japan. (Weak recommendation, Evidence level B, 100% agreed).

*Comment*: The combined findings of case–control and cohort studies demonstrated that PPI [122], aspirin [123], COX inhibitors [124], and statins (HMG-CoA inhibitors) [125] may effectively prevent carcinogenesis in esophageal adenocarcinoma with Barrett’s epithelium. However, due to large variations in background factors between groups and the quality of the original studies, there has been no consensus on COX inhibitors, including aspirin and statins, and evidence is considered to be insufficient [126]. On the other hand, there have been several relatively large cohort studies on PPI, and based on the findings of a meta-analysis by Singh et al. [122], PPI may reduce the incidence of high-grade dysplasia (HGD) and esophageal adenocarcinoma from BE. A RCT was recently performed [127]. In this study, 2557 BE patients at 84 centers in the UK and Canada were randomly assigned to 4 groups: high-dose (80 mg/day) or low-dose (20 mg/day) esomeprazole and aspirin (300 or 325 mg/day). There were 313 primary endpoint events (all-cause mortality, esophageal adenocarcinoma, or HGD) with a median follow-up of 8.9 years. The high-dose PPI group significantly reduced the composite primary endpoint events compared to the low-dose PPI group, whereas no significant differences were observed in the groups treated with and without aspirin. However, these findings included total mortality, and its effect on the incidence of esophageal adenocarcinoma/HGD was not significant; however, the incidence of esophageal adenocarcinoma/HGD was slightly lower in the high-dose PPI group. Since the high-dose PPI used in this study was esomeprazole at 80 mg/day, which is four times the standard dose in Japan, and no similar study has been performed in Japan, the recommendation is “It is proposed that medication for the prevention of carcinogenesis of BE is not currently be used in Japan.”

## Appendix

The members of the Guidelines Committee who created and evaluated the Japanese Society of Gastroenterology ‘‘Evidence-based clinical guidelines for gastroesophageal reflux disease’’ are listed below.

### Creation Committee

Chair: Katsuhiko Iwakiri (Department of Gatroenterology, Nippon Medical School Graduate School of Medicine). Vice-Chair: Yasuhiro Fujiwara (Department of Gastroenterology, Osaka City University, Graduate School of Medicine).

Members: Noriaki Manabe (Division of Endoscopy and Ultrasonography, Department of Clinical Pathology and Laboratory Medicine, Kawasaki Medical School), Eikichi Ihara (Department of Gastroenterology and Metabolism, Graduate School of Medical Sciences, Kyushu University), Shiko Kuribayashi (Department of Gastroenterology and Hepatology, Gunma University Graduate School of Medicine), Junichi Akiyama (Department of Gastroenterology, National Center for Global Health and Medicine), Takashi Kondo (Division of Gastroenterology and Hepatology, Department of Internal Medicine, Hyogo College of Medicine), Hiroshi Yamashita (Sugimoto Kenji Clinic), Norihisa Ishimura (Second Department of Internal Medicine, Shimane University Faculty of Medicine), Yuichi Kitasako (Dental Clinic, Ministry of Foreign Affairs), Katsunori Iijima (Department of Gastroenterology, Akita University Graduate School of Medicine), Tomoyuki Koike (Division of Gastroenterology, Tohoku University Graduate School of Medicine), Nobuo Omura (National Hospital Organization, Nishisaitama-Chuo National Hospital), Tsutomu Nomura (Department of Gastrointestinal and Hepato-Billiary-Pancreatic Surgery, Nippon Medical School).

### Evaluation Committee

Chair: Yoshikazu Kinoshita (Steel Memorial Hirohata Hospital). Vice-Chair: Shuichi Ohara (Tohoku Rosai Hospital).

Members: Soji Ozawa (Tamakyuryo Hospital), Osamu Kawamura (Kamimoku SPA Hospital).

### Japanese Society of Gastroenterology

President: Kazuhiko Koike (Kanto Central Hospital). Past President: Tooru Shimosegawa (South Miyagi Medical Center). Directors Responsible: Satoshi Mochida (Department of Gastroenterology & Hepatology, Saitama Medical University), Nobuyuki Enomoto (First Department of Internal Medicine, Faculty of Medicine, University of Yamanashi).
